# Specific Interaction With Human Serum Albumin Reduces Ginsenoside Cytotoxicity in Human Umbilical Vein Endothelial Cells

**DOI:** 10.3389/fphar.2020.00498

**Published:** 2020-04-29

**Authors:** He Li, Chen Chen, Zhong-Ming Li, Yang Yang, Chao-Qun Xing, Yang Li, Ying-Hua Jin

**Affiliations:** ^1^Key Laboratory for Molecular Enzymology and Engineering, the Ministry of Education, College of Life Science, Jilin University, Changchun, China; ^2^The First Affiliated Hospital to Changchun University of Chinese Medicine, Changchun, China

**Keywords:** albumin, cytotoxicity, ginsenoside, human vascular endothelial cells, isothermal titration calorimetry, molecular docking

## Abstract

Human serum albumin (HSA) is an important component of plasma, which has the functions of maintaining colloid osmotic pressure and capillary membrane stability, promoting blood circulation, and anti-oxidation. Three-dimensional structure of HSA determines its ability to bind and transport hormones and other substances. In this study we examined the interactions between HSA and ginsenoside Rg3, Rg5, Rk1, Rh2, and Rh4, which are the main cytotoxic ginsenosides extracted from red ginseng. Heat transfer generated by the specific interaction between HSA and each ginsenoside was measured using isothermal titration calorimetry (ITC) assay, which demonstrated that all these 5 ginsenosides bound to HSA with binding constants of 3.25, 1.89, 6.04, 2.07, and 5.17 × 10^5^ M^−1^, respectively. Molecular docking also displayed that these ginsenosides interact with HSA at different sites of the HSA surface. Importantly, cell viability assay showed that the cytotoxicity of these ginsenosides reduced significantly at the presence of HSA in human vascular endothelial cells (HUVEC). Taken together, this study reveals the mechanism by which these ginsenosides are transported *in vivo* by not causing damage in vascular endothelium, and also suggests HSA might be an ideal carrier help to transport and execute these ginsenoside functions in human body.

## Introduction

Ginseng, a valuable Chinese herb, has been used in China, Korea, and other oriental countries for thousands of years (Lin et al., 2015). In recent years, a large number of studies have demonstrated its efficacy in improving immunity (Tran et al., 2014), enhancing memory (Zhang et al., 2008), improving cardiovascular function (Li-Ping et al., 2016; Zheng et al., 2017), anti-tumor (Lin et al., 2015), and delaying aging (Zhou et al., 2015; Wang et al., 2018). Ginsenoside, a kind of glycoside compounds composed of glycogen and glucose, is widely regarded as the main active ingredient of ginseng (Kim et al., 2015), because they have its own function similar to ginseng’s pharmaceutical function, such as anti-inflammation (Baek et al., 2015; Yang et al., 2015), anti-tumor, and anti-aging effects, which have been shown in a series of studies *in vitro* and *in vivo*.

Most ginsenosides have low bioavailability (Yang et al., 2011). After ingestion, some ginsenosides, such as Rb1, the most abundant ginsenoside, are metabolized into smaller molecules in the gastrointestinal tract through hydrolysis and then absorbed by the intestine (Kim et al., 2013). Most ginsenosides were directly discharged from the body due to their high molecular weight and poor transmittance of mucous membrane (Akao, 1998; Paek et al., 2010). Meanwhile, studies on the tumor cell cytotoxicity of ginsenosides have also increased over the years (Tang et al., 2017; Wu et al., 2017), for example, by activating apoptosis pathways or targeting NF-κB pathway to inhibit cell growth (Wang et al., 2018). Thus, it is largely unknown whether the tumor cell cytotoxic ginsenosides also inhibit normal cell growth and cause damage *in vivo*.

Human serum albumin (HSA), a product of liver, is an important component of blood (Roche et al., 2008). The concentration of HSA in human blood is about 40 mg/mL, accounting for 60% of the total protein content in blood (Squella et al., 1987). HSA has the functions of maintaining colloid osmotic pressure, stabilizing capillary membrane (Margarson and Soni, 1998), anti-oxidation (Roche et al., 2008), and promoting blood circulation (Evans, 2015). Because of the structural characteristic of HSA, it can bind and transport a variety of substances, including cholesterol, fatty acids, hormones, bilirubin, and various anions. Our previous work has shown that HSA binds to ginsenoside Rh2(G-Rh2) and reduces its cytotoxicity (Lin et al., 2017). Here we show that HSA also binds to a series of anticancer cell ginsenosides including Rg3, Rg5, Rk1, and Rh4, which extracted from red ginseng, and the binding with HSA totally inhibited their cytotoxicity in HUVEC cells.

## Materials and Methods

### Materials

Ginsenoside Rg3, Rg5, Rk1, Rh2, Rh4, and compound K were purchased from Sigma-Aldrich (≥98% HPLC, St. Louis, MO, USA). Recombinant human serum albumin was purchased from Healthgen Biltechnology Ltd. (Wuhan, China). 3-(4,5-dimethylthiazol-2-yl)-2,5-diphenyltetrazolium bromide (MTT) and dimethyl sulfoxide (DMSO) were purchased from Sigma-Aldrich (St. Louis, MO, USA). Fetal calf serum was purchased from Biological Industries (Kibbutz Beit-Haemerk, 25115, Israel). Dulbecco modified Eagle’s medium (DMEM) was purchased from Gibco BRL (Grand Island, NE, USA). The following primary antibodies was used in this research:rabbit anti-PARP (Santa Cruz) and rabbit anti-cleaved caspase 3 (Cell-Signaling). The secondary antibody HRP-conjugated goat anti-rabbit IgG was purchased from Pierce (Thermal Scientific).HUVEC cells were obtained from the American Type Culture Collection (ATCC, Rockville, MA, USA).

### Methods

#### ITC Assay

In this research, interaction between HSA and ginsenosides were identified with isothermal titration calorimetry on Malvern MicroCal ITC200. 40 μL HSA (400 μM) was titrated into 200 μL different ginsenosides (20 μM) in the sample cell independently. Data of titrations was collected by MicroCal ITC200 system (MAN0560-02-EN-00, May 2015), and MicroCal ITC-ORIGIN Analysis Software (MAN0577-02-EN-00, May 2015) was used for data processing. All the titrations were carried out at room temperature.

#### Molecular Docking Analysis

Molecular docking simulation were carried out by using AutoDock software (version 4.2.6) based on Lamarckian Genetic Algorithm (Scripps Research Institute, La Jolla, CA, USA) under default settings. Structures of ginsenosides were optimized by Chem3D 16.0 software to minimize their energy. Three-dimensional structure of ginsenosides were downloaded from NCBI Pubchem Compound database (Pubchem ID: Rg3, 9918693; Rg5, 1155001; Rk1, 11499198; Rh2, 119307; Rh4, 21599928.), and the crystal structure of human serum albumin was downloaded from RCSB Protein Data Bank (PDB ID: 1AO6). Results were optimized according to the empirical scoring function which estimates the binding free energy of the predicted complexes and visualized by PyMOL software (ver. 0.99, DeLano Scientific LLC).

#### Cell Culture

HUVEC cells were grown in DMEM with 10% v/v fatal calf serum, 100 μg/mL of streptomycin, and 100 U/mL of penicillin at 37°C in CO_2_ incubator with 5% CO_2_ in saturated humidity.

#### Cell Viability Assay

Exponentially growing HUVEC cells were resuspended in DMEM with 10% fetal calf serum and seeded into a 96-well plate at 1×10^4^ cells per well. After incubation at 37°C with 5% CO_2_, the culture medium were exchanged to DMEM without serum, then cells were treated with different ginsenosides in concentration from 0 to 50 μg/mL for 48 h in triplicate. At the 44th hour, 20 μL of MTT (5 mg/mL, Sigma) was added into each well, then incubated with cells for 4 h. All the culture medium were removed and 150 μL DMSO was added into each well to solubilize the formazan crystals formed by viable cells. Absorption at 550 nm of each well was measured with a microplate reader (TECAN, Maennedorf, Switzerland).

### Statistical Analysis

Data were presented as mean ± standard deviation with Microsoft Office 2013 and imaged with Sigma plot 10 (Systat Software Inc., San Jose, CA). Statistical significance was calculated with Student’s *t*-test. Differences were considered statistically significant as follow: *p < 0.05; **p < 0.01; ***p < 0.001.

## Results

### HSA Interacts With a Series of Ginsenosides

**Figure 1:**
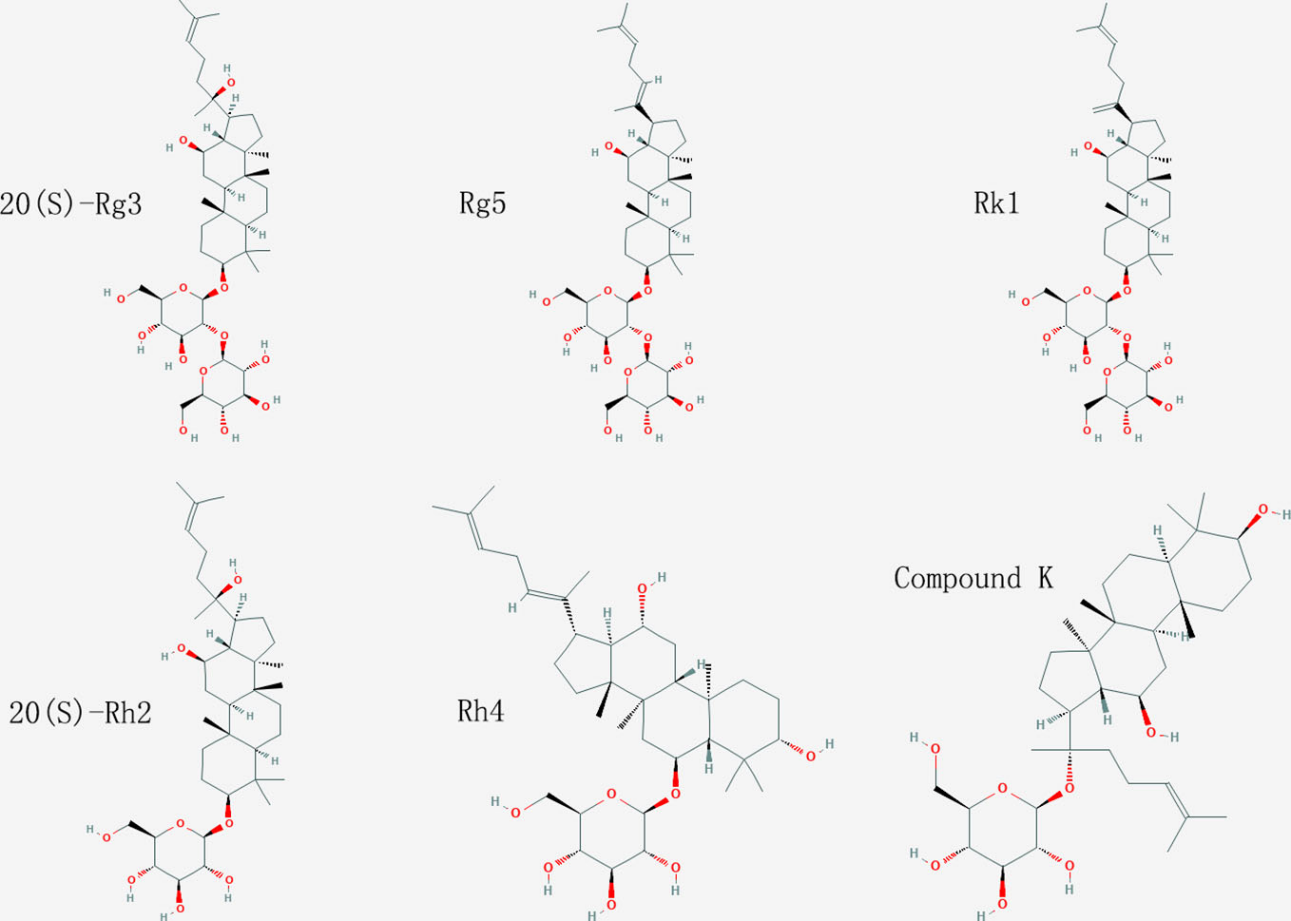
Chemical structure of ginsenosides used in this study.

**Figure 2:**
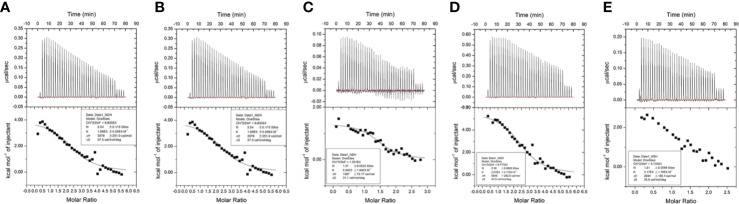
Results of isothermal titration calorimetry (ITC) assay. **(A)** (20S)G-Rg3. **(B)** G-Rg5. **(C)** G-Rk1. **(D)** (20S)G-Rh2. **(E)** G-Rh4.

Isothermal titration calorimetry (ITC) assay is a well-established method used in quantitative studies of biomolecule interactions. When the binding between protein and small molecular compound occurs, heat will be either absorbed or released. Heat transfer is measured by calorimeter during the titration, and this measuring enables accurate determination of binding constants, reaction stoichiometry, enthalpy, and entropy. In this study, we determined the interaction of ginsenoside Rg3, Rg5, Rk1, Rh4, and Rh2 (chemical structures showed in **Figure 1**) with HSA by ITC. All the binding of these ginsenosides and HSA were endothermic, and the binding constant of ginsenoside Rg3, Rg5, Rk1, Rh4, and Rh2 with HSA was 3.25, 1.89, 6.04, 2.07, and 5.17×10^5^ M^−1^, respectively. Parameters of titration were shown in **Figure 2**.

### HSA Interact With Ginsenosides Through Different Binding Sites

**Figure 3:**
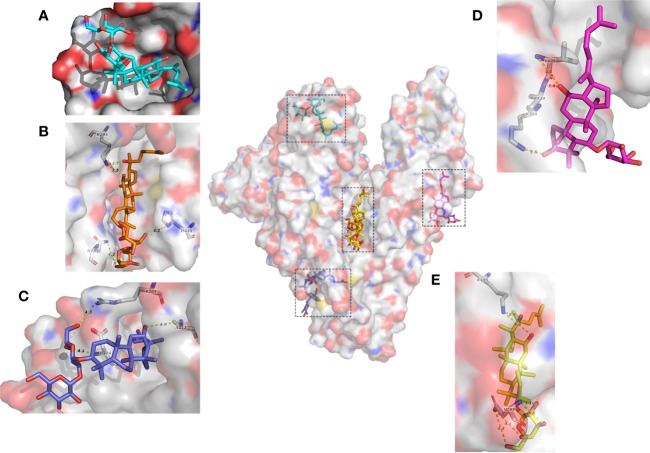
Molecular mechanisms of interactions between HSA and ginsenosides. **(A)** G-Rk1. **(B)** (20S)G-Rg3. **(C)** G-Rg5. **(D)** G-Rh4. **(E)** (20S)G-Rh2.

Next we investigated the molecular mechanism of the interaction between HSA and ginsenoside Rg3, Rg5, Rk1, Rh4, and Rh2 by molecular docking. 10 results obtained by using Autodock software in each simulation with different binding sites or conformations. In accordance with the binding constant from the ITC assay, we selected the solution closest to the real situation among all the results and analyzed key residues at the binding sites (**Figure 3**).

Interestingly, even if these ginsenosides have similar chemical structures, each ginsenoside binds to different sites of HSA with different molecular forces, and Rg3, Rg5, Rh2, and Rh4 interact with HSA mainly by electrostatic interaction, while Rk1 interacts completely by hydrophobic interaction. Visualization of these results was shown in **Figure 3**.

### HSA Reduces the Cytotoxicity of Ginsenosides in HUVEC Cells

**Figure 4:**
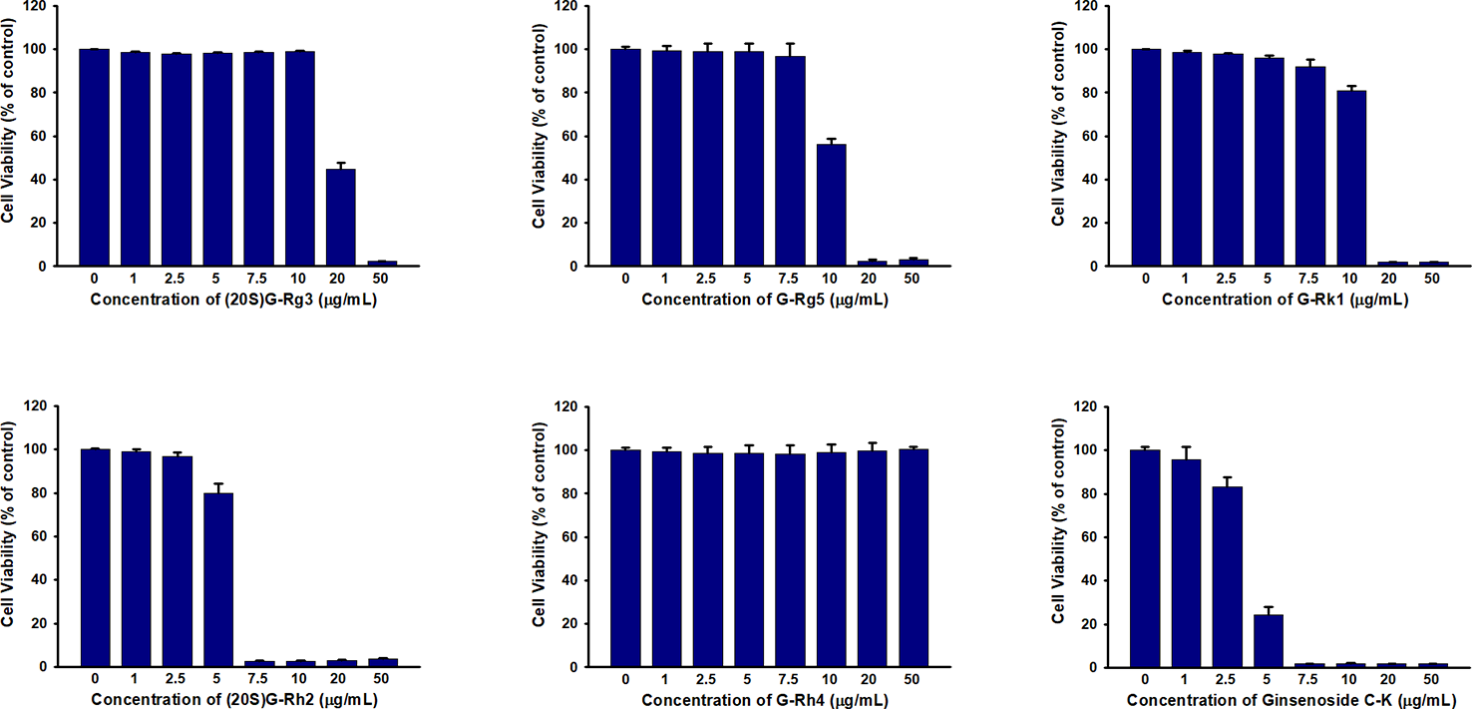
Cell viability assay of HUVEC treated with ginsenosides Rg3, Rg5, Rk1, Rh4, Rh2, and Compound K.

**Figure 5:**
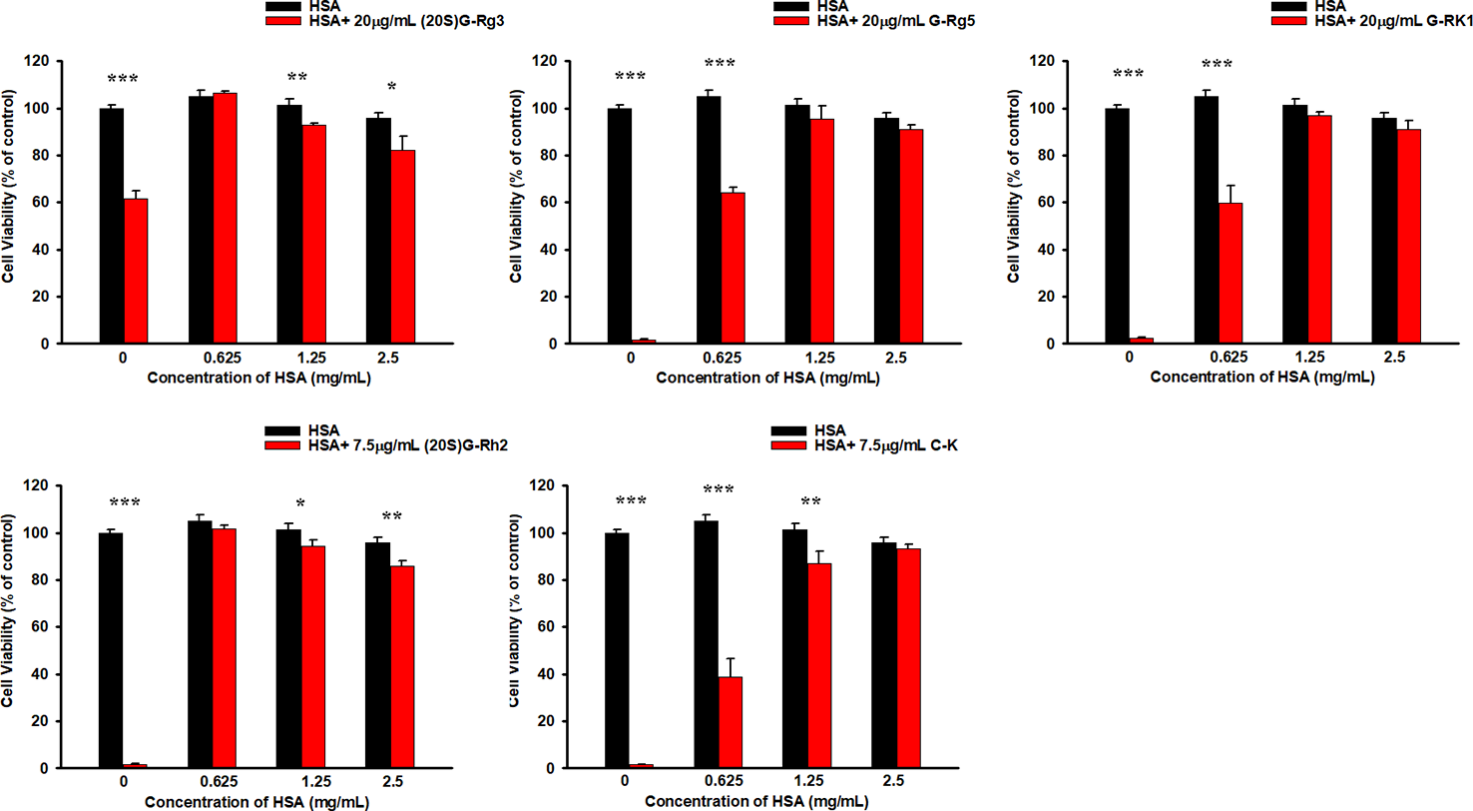
HSA reduced the cytotoxicity of ginsenosides on HUVEC cells. Differences were considered statistically significant as follow: *p < 0.05; **p < 0.01; ***p < 0.001.

In previous studies we have demonstrated that HSA and BSA reduced G-Rh2 cytotoxicity in human hepatoma HepG2 cells by binding to it. However, it is not clear whether cytotoxic ginsenosides make damage in HUVEC cells. We first examined the effect of ginsenosides used in ITC assay and compound K on cell viability of HUVEC cell by MTT assay. Results showed that all the 5 ginsenosides except Rh4 exhibit cell growth inhibitory effect in HUVEC cells which shown in **Figure 4** and **Table 1**. To examine the HSA effect on the cytotoxicity of these ginsenosides in HUVEC cells, we determined cell viability at the presence of increasing concentrations of HSA. The results showed that HSA, even at low concentration (1.25 mg/ml) significantly reduced ginsenoside cytotoxicity in HUVEC cells (**Figure 5**).

**Table 1:** IC_50_ of different ginsenosides to HUVEC cells.

Ginsenoside	(20S)G-Rg3	G-Rg5	G-Rk1	(20S)G-Rh2	G-Rh4	Compound K
IC_50_ (μg/ml)	19.576 ± 0.250	10.182 ± 0.292	12.272 ± 0.341	5.283 ± 0.048	–	4.107 ± 0.667

## Discussion

Ginsenosides are the main active components of ginseng. In recent years, researchers have begun to study ginsenoside by means of cell biology, focusing on their functions of anti-inflammation, inhibiting tumor cell proliferation, migration, and activating apoptosis pathways. Most of these studies were conducted *in vitro*. In fact, when ginsenosides are ingested through diet, due to their poor bioavailability and easy degradation in the digestive tract, we cannot guarantee whether ginsenosides will be eventually transported to the places where they are needed. Previous studies have shown that some ginsenosides are absorbed by small intestinal villi through passive diffusion (Paek et al., 2010), and ginsenosides used in this study have a considerable bioavailability and stability *in vivo* (Zhu et al., 2018). Here we demonstrated the interactions between 5 sort of ginsenosides from red ginseng and HSA by ITC and molecular docking. These results indicates that these ginsenosides were transported through the bloodstream to various parts of the body by binding with HSA after absorption by intestine, and also the interaction with HSA may result in the strong stabilities of these ginsenosides *in vivo* demonstrated in previous study (Zhu et al., 2018).

Because of the cavities and depressions on the surface of HSA molecule, it can transport many substances by interacting with them. Amounts of drug molecules can bind to HSA reversibly, with binding constants ranging from 1×10^4^ to 1×10^6^ M^−1^ (Kragh-Hasen et al., 2002; Colmenarejo, 2010). In present study we found that ginsenosides with similar structures including ginsenoside Rg3, Rg5, Rk1, Rh4, and Rh2 can bind to HSA at different sites, indicating the binding with HSA might be a universal property of ginsenosides.

A number of *in vitro* studies have shown that ginsenoside Rg3, Rg5, Rk1, Rh2, and compound K lead to cell death in various tumor cells, generating a possibility that these reagents could be developed as new cancer drugs. As a drug candidate, it should not cause damage to vascular endothelia cells. Our previous study have shown that HSA even at a low concentration (1.68 mg/mL) effectively reduced cell viability by about 50% (Lin et al., 2017). However, only 0.625 mg/mL of HSA totally protected HUVEC cell damage induced by G-Rh2. These data suggested that tumor cells and normal cells might have different tolerance levels to ginsenosides, and it might be caused by different status between tumor cells and normal cells, such as differences in metabolic pathways, ROS levels (Liou and Storz, 2010), the expression level of ginsenoside-targeting proteins (Wang et al., 2017), and the integrity of apoptosis and/or autophagy signaling pathways. The possibility and the exact molecular and cellular mechanism that tumor cells are more sensitive to certain ginsenoside should be studied in future research.

## Data Availability Statement

All datasets generated for this study are included in the article/Supplementary Material.

## Author Contributions

Y-HJ and YL supervised the research. HL and Z-ML performed the isothermal titration calorimetry (ITC) assay. CC performed the molecular docking. YL, YY, and C-QX performed the cell viability assay. Y-HJ and HL wrote the manuscript.

## Funding

This study was funded by Special Project for Province & University Construction Plan of Jilin Province (SXGJXX2017-13) and Science and technology development program of Jilin Province (20191102059YY).

## Conflict of Interest

The authors declare that the research was conducted in the absence of any commercial or financial relationships that could be construed as a potential conflict of interest.
